# Plant and Soil Core Mycobiomes in a Two-Year Sorghum–Legume Intercropping System of Underutilized Crops in South Africa

**DOI:** 10.3390/microorganisms10102079

**Published:** 2022-10-20

**Authors:** Gilmore T. Pambuka, Tonjock Rosemary Kinge, Soumya Ghosh, Errol D. Cason, Martin M. Nyaga, Marieka Gryzenhout

**Affiliations:** 1 Department of Genetics, University of the Free State, Bloemfontein 9301, South Africa; 2 Department of Biological Sciences, University of Bamenda, North-West Region, Cameroon; 3 Department of Animal Sciences, University of the Free State, Bloemfontein 9301, South Africa; 4 Next Generation Sequencing Unit, Department of Biological Sciences, Division of Virology, University of the Free State, Bloemfontein 9301, South Africa

**Keywords:** core mycobiomes, sorghum, legumes, next-generation sequencing, intercrop, substrates

## Abstract

Fungal communities form close beneficial (mutualists) or detrimental (pathogens) associations with their plant hosts. Their diversity and abundance can be affected by agricultural practices which include cropping systems such as rotations and intercropping. Despite the importance of cropping systems in increasing productivity, knowledge of the fungal mycobiome and the core inhabitants for under-utilised cereal and legume crops, particularly over a period, is still limited. The core mycobiomes in plant tissues and bulk soils of a cereal–legume intercrop were characterized over two years using high-throughput sequencing. The intercropping trial consisted of sorghum, Bambara groundnut, cowpea, dry bean, and soybean. A greater number of molecular operational taxonomic units (MOTUs) were found in plant tissues compared to those from the soils and between year one and year two. Principal coordinate analyses revealed that fungal communities for each year were relatively distinct, particularly for the soils. The core mycobiome was dominated by a Davidiellaceae sp. (*Cladosporium*), Didymellaceae sp. 1 (*Phoma*), Didymellaceae sp. 2 (*Epicoccum*), *Fusarium* sp. 2, Unidentified (Ascomycota), and *Cryptococcus* MOTUs that were present in all plant tissues and soils of year one and two. Other key MOTUs were only specific to a year, substrate, or crop. Although the mycobiome of sorghum were more distinct than the cores of the legumes, there were still MOTUs dominant across all of the crops. Characterization of this baseline core across two years provides insight into those fungi that are always present in these crops, and that could be utilized in improving crop performance and productivity.

## 1. Introduction

Globally, there has been a reduction in arable land suitable for crop production [1]. With the pressures of growing populations and threats on food security, alternative systems have been adopted to intensify and increase agricultural production as well as to mitigate other factors, such as climate change, that have impacted agriculture heavily [2]. Intercropping is the practice of growing two or more crops in the same environment and has been recognised as a feasible and stable agricultural system that exhibits greater performance and resilience than sole-cropping systems (monoculture) [3,4]. Unlike sole-cropping systems, intercropping improves soil fertility and yield, reduces disease and pest incidences, and provides good financial returns [4]. This is particularly true for subsistence farming, for which it also provides diversification in terms of diet and reduces the risk of complete failure of one crop type due to changes in climatic conditions or diseases and pests [4].

The potential of underutilised crops in contributing to food security has been highlighted in literature [5,6]. Most of the underutilised crops have attributes such as drought and heat stress tolerance and resistance to pests and diseases, making them ideal for production in low-input agricultural systems and in semi-arid and arid areas [5,6]. In addition, some of the underutilised crops are known for their high nutrient value, which is ideal for diet diversification and addressing nutrient deficiencies, especially in poor rural communities [7]. However, in South Africa, underutilised crop improvement and development is still largely limited compared to those of several major commercial crops, such as maize, wheat, and soybean [8].

Sorghum (*Sorghum bicolor*) is an important major cereal crop grown mostly in semi-arid regions of Africa and Asia as a source of food and fodder, particularly in smallholder farming communities [9]. Globally and in South Africa, soybean (*Glycine max*) and dry bean (*Phaseolus vulgaris*) are of commercial importance mainly for human and animal consumption [10,11]. Cowpea (*Vigna unguiculata*) serves as a dual-purpose legume crop for human consumption and livestock fodder and is still considered an underutilised crop [12,13]. These legumes have been considered as important intercropping plants with positive effects on diversified cropping systems [14,15]. Bambara groundnut (*Vigna subterranea*) is an under-utilised legume crop grown for its seed on lower scales in sub-Saharan Africa by rural farmers as a staple food [5].

The phytobiome consists of plants, their environment, and interacting micro- and macro-organisms [16]. Micro-organisms associated with the plant make up the plant microbiome, which includes bacteria, fungi, viruses, and archaea [17]. Plant species have been reported to maintain a core microbiome consisting of microbes consistently associated with the crop. Core microbiomes include common cosmopolitan species and microbial members with more specific functional relationships with their host [18,19]. The concept of core microbiomes was first introduced for human microbiomes and later expanded into identifying core plant microbiomes [19].

The definition and concept of core microbiomes is based on species abundance, occupancy, or both in different plant species and niches [18,20]. The core microbiome in plants has been applied in various contexts based on different research methods. Some studies looked at the core microbiome in different plant species based on taxon minimum occupancy frequency thresholds and the proportions and detection of species and molecular operational taxonomic units (MOTUs) across samples [18,20,21,22]. Other definitions include [21], who defined the core microbiome as genera or species with at least 90% occupancy in analysed samples or as microbes occupying all the samples under consideration, regardless of their abundance [22]. Others defined the core microbiome as those taxa consistently detected on a plant host or environment [18,23]. More studies looked at abundant microbial taxa ranked by relative abundance percentage tables, graphs, and curves [18,20,24].

Assessments for core mycobiomes are performed despite factors such as the environment, soil type, plant genotype, and agricultural management practices that can affect the diversity and composition of microbial communities [18,23]. For example, [23] reported a consistent rhizosphere core microbiome for wheat despite differences in soil characteristics. The composition of core plant microbiomes has already been studied for several plants in different habitats, geographical environments, and ecosystems. These include rice (*Oryza sativa*), wheat (*Triticum aestivum*), *Arabidopsis thaliana*, and danshen (*Salvia miltiorrhiza*) [22,23,24,25,26].

The mycobiome is the fungal component of the phytobiome of a plant and represents a group diverse in ecological functionalities, including plant growth promoters, decomposers, pathogens, and mutualists [27]. Mycobiomes of crops have been poorly studied in Africa. The aim of this study is to investigate the core fungal mycobiomes in plant tissues and soils of sorghum and four legumes (Bambara groundnut, soybean, cowpea, and dry bean) planted in an intercropping trial over a two-year period in the same field using targeted Illumina sequencing of the fungal internal transcribed spacer (ITS) 2 region. This is the first time a core fungal mycobiome has been established in such a system. Understanding the core mycobiome of these crops and the bulk soils surrounding them will aid in establishing the fungi that will most likely always be associated with these crops. Knowledge gained could help in promoting sustainable cropping systems for plant health and productivity.

## 2. Materials and Methods

### 2.1. Field Sampling

The trial plan (Supplementary Figure S1) was planted at the Grain Crops Institute, Agricultural Research Council in Potchefstroom, South Africa. Asymptomatic sorghum and legume plants for environmental sequencing were sampled in the 2015/2016 season for year one, while year two plants were sampled in the 2016/2017 season. For each year, a total of 5 random whole plants with soils still adhering to roots and bulk soil from the middle of two adjacent rows were uprooted from the ground for each of three repeats per plant species (Supplementary Figure S1), thus totalling 15 plant and 15 soil samples per crop. Year one consisted of a single sorghum cultivar, PAN8816, intercropped with cowpea, soybean, Bambara groundnut, dry bean, and fallow soils. The trial layout for year two was similar except that three sorghum cultivars, PAN8076W, PAN8816, and NS5511, were planted with the legumes. Sorghum and legume plant material and their soils were transported cold in large plastic and zip lock bags to the laboratory of the Department of Genetics, University of the Free State for further processing.

### 2.2. Processing of Plant Material and Soils

Plant material from sorghum, Bambara groundnut, dry bean, cowpea, and soybean in year one and two were cut and separated into above-ground parts (seed, leaves, stems) and below-ground parts (roots). The plant material for each crop was chopped into pieces, followed by a thorough wash to remove dust and soil debris. Plant parts for each crop were surface-sterilized via sequential washing with 3% sodium hypochlorite for 3 min followed by sterile distilled water for 1 min, 70% ethanol for 2 min, and a final wash with distilled water for 1 min. After surface sterilization, plant parts were air-dried and placed into 50 mL falcon tubes. The bulk soil samples (20 g) from each crop were transferred into 25 mL falcon tubes. All plant material and bulk soils were freeze dried and ground using a home mince grinder (thoroughly surface-sterilized between samples). Representative amounts (20 g) of the ground samples per treatment and soils were collected, transferred into 2 mL Eppendorf tubes, and pulverized in a Qiagen Tissue Lyser II cell disrupter (Whitehead Scientific, Cape Town, South Africa) for DNA extractions.

### 2.3. DNA Extraction and Illumina Sequencing

Total genomic DNA was extracted from 0.1 g of each pulverized plant tissue sample per plant, and 0.5 g of soil using the + (Macherey Nagel, Dueren, Germany) and the Nucleospin^®^ Soil kit (Macherey Nagel, Germany). DNA concentration and quality were checked using a Nanodrop LITE spectrophotometer (Thermo Fisher Scientific, Waltham, MA, USA) and diluted to 10 ng/µL (1:10). For the polymerase chain reactions (PCR), the internal transcribed spacer 2 region (ITS 2) was amplified using the ITS3F (5′-GCATCGATGAAGAACGCAGC-3′) and ITS4R (5′-TCCTCCGCTTATTGATATGC-3′) primer set with over-hanging Illumina adapters [28]. The PCR reactions consisted of a final volume of 25 µL mixture containing 12.5 µL Kapa HiFi Ready-Mix DNA Polymerase (KAPA Biosystems, Lasec, South Africa), 1.5 µL 10 mM ITS3F and ITS4R primers, 9 µL nuclease-free water, and 2 µL template DNA. The PCRs were performed using the G-Storm GS04822 thermal cycler (Somerton Biotechnology Centre, United Kingdom) at 3 min for initial denaturation at 95 °C followed by 25 cycles at 95 °C denaturation for 30 s, annealing at 58 °C for 30 s, extension at 72 °C for 30 s, and a final extension at 72 °C for 5 min. The PCR products were visualized under a UV light via 2% agarose gel electrophoresis with GelRed (Biotium, Inc., Fremont, CA, USA) fluorescent nucleic acid dye.

The PCR amplicons were sent for Illumina sequencing preparations at the Next Generation Sequencing Unit, Health Sciences, University of the Free State, South Africa. Purification of the PCR amplicons was performed using the Agencourt AMPure XP bead clean-up kit (Beckman Coulter, Atlanta, GA, USA), followed by quantification of the final library using a Qubit 3.0 fluorometer (Life Technologies, Thermo Fisher Scientific) and validation using the Bioanalyzer 2100 (Agilent Technologies, Santa Clara, CA, USA) to verify fragment size (200–300 bp). Purified amplicons were normalized and pooled together for paired-end sequencing (2 × 300 bp) using a MiSeq V3 (600 cycle) kit (Illumina Inc., San Diego, CA, USA) on an Illumina MiSeq platform (Illumina Inc., San Diego, CA, USA).

### 2.4. Cluster and Data Analysis

Sequenced data for both years followed an analysis workflow using various Bioinformatics software [29]. Pre-processing and quality checks of forward and reverse sequences were assessed using FastQC v 0.11.8- Babraham Bioinformatics [30]. Prinseq lite version V0.20.4 was used for the trimming and quality control of sequences to obtain an average quality score of ≥25 and a minimum sequence length of 200 bp [31]. Default parameters in PEAR 0.9.6 were used to merge paired-end sequences [32]. QIIME v1.9.1 was used to analyse paired-end reads [33]. Chimeric sequence identification was conducted using USEARCH 6.1 [34] against the RDP “Gold” database and was filtered with QIIME using the identify_chimeric_seqs.py and filter_fasta.py commands. Rare reads were excluded if they occurred only once. Sequence clustering and assignment into molecular operational taxonomic units (MOTUs) was performed using the pick_open_reference_otus.py script against the ITS UNITE database (alpha version 12_11) released on 10.10.2017 [35], at a similarity threshold of 97% [36]. In some cases, it is known that ITS sequence data cannot be confidently used to identify to species level, e.g., *Cladosporium*, *Phoma,* and *Epicoccum* [29]. MOTUs were only referred to on the family level. MOTUs named by the pipeline with synonymous names—such as *Giberrella,* currently known under *Fusarium*—were changed to the current name with the distinction indicated as sp. “x”, with x being a number based on the number of MOTUs for that genus [37].

QIIME version 1.9.1 was used to normalize the OTU table using the CSS normalization option [38]. Data generated for all plant parts for sorghum and legumes in year two were combined to give total plant tissue for each crop. Soils were treated separately. Data from year-one and -two crops were combined in QIIME 1 using the command merge_otu_tables.py. For comparison and analysis, only data from the sorghum PAN8816 cultivar were used, and year-one and -two fungal alpha diversities (i.e., abundance and richness) were calculated using the Observed OTU indices and Shannon diversity metrics using the command alpha_rarefaction.py. Beta diversity was performed using Bray–Curtis dissimilarity metrics and visualized with principal coordinates analysis (PCoA) plots in RStudio [39] using the “plot_ordination” function in the “Phyloseq” package [40]. The software was used for additional analyses to visualize fungal diversity indices in different samples (rarefaction curves and bar charts). The statistical significances of detected differences between plant niches and year were compared using permutational multivariate analysis of variance (PERMANOVA) using the function “adonis2” in the vegan package [37] Unidentified MOTUs were not discarded and were included in the analysis. The core fungal communities for plant tissues and soils from year one and two were based on the concepts used by [22], which required that genera must be present in all samples to be part of the core. Names allocated by the pipeline were moderated in cases in which they could be misleading, such as when it is known that the sequenced region cannot distinguish between species or genera, as is the case with the Didymellaceae and Mycosphaerellaceae, or in the case of older names, such as *Gibberella* for *Fusarium* [27,29]. The tables incorporated for RA showed less diversity; therefore, Venn diagrams were plotted using the function in gplots in RStudio (https://CRAN.R-project.org/package=gplots, accessed on 25 June 2021) to show the total diversity between plant niche and year. Sequence data will be submitted to the Bioprojects of Genbank as PRJNA882429.

## 3. Results

### 3.1. Illumina Sequencing and Data Analysis

After quality control, the average sequence length ranged from 251 to 300 bp at a base Phred quality score >25. Sequence numbers which were retained for the different crops in each year ranged from 2098 to 9470 (year one) and 165,487 to 845,135 (year two) in plant tissues and 77,709 to 139,545 (year one) and 57,655 to 77,709 (year two) in soils (Table 1). The sequences in each year were represented by individual MOTUs per crop ranging from 60 to 87 MOTUs (year one) and 185 to 229 MOTUs (year two) in plant tissues. In the soils, the number of MOTUs ranged from 277 to 747 MOTUs (year one) and 148 to 272 MOTUs (year two). Rarefaction curves for both years indicated that there was possible underestimation of fungal diversity in some samples and that deeper sequencing is required to completely resolve community diversity (Supplementary Figure S2).

### 3.2. The Core Mycobiome across All Crops, Substrates, and Years

The overall core mycobiomes across crops, substrates and years consisted of a Davidiellaceae sp. (*Cladosporium*), Didymellaceae sp. 1 (*Phoma*), Didymellaceae sp. 2 (*Epicoccum*), *Fusarium* sp. 2, an unclassified MOTU (Ascomycota), *Cryptococcus*, and an unidentified MOTU (Table 2). Among these, the core MOTUs dominant across years, substrate, and crops included Didymellaceae sp. 1 (*Phoma*), *Fusarium* sp. 2, and Unidentified (Ascomycota). The core MOTUs varied in frequency and distribution of RA between the years, while other MOTUs, such as Didymellaceae sp. 2 (*Epicoccum*), remained relatively the same. For example, Davidiellaceae sp. (*Cladosporium*), Didymellaceae sp. 1 (*Phoma*) and Unidentified (Ascomycota) decreased in Year 2, while *Cryptococcus* increased.

**Figure 1:**
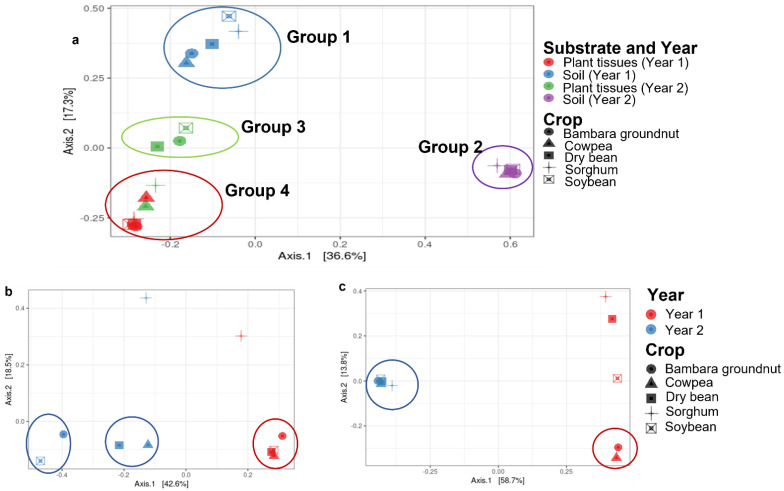
Principal coordinate analysis (PCoA) for the fungal communities from plant tissue and soils of sorghum and legumes over two years. All substrates (**a**), plant tissues (**b**), and soil (**c**).

Fungal mycobiomes formed distinct communities and were statistically significant (*p* = 0.001), based on year and substrate (plant niche) when all data were grouped (Figure 1). Soils from year two were most distant, while MOTUS from plant material in year one overlapped with those from year two. No overlap was found between soils and plants.

### 3.3. The Core Mycobiomes of Crops

**Figure 2:**
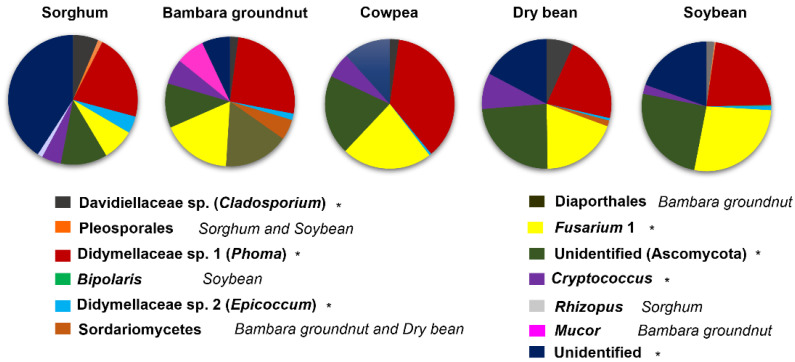
Fungal diversity of the core mycobiome. The pie chart illustrates the diversity richness of fungal MOTUs per crop. MOTUs with an asterisk (*) indicate the core across crops.

Overall, the crops had the same core mycobiomes, albeit with variations in RA and occurrence specific to a crop or year (Figure 2; Supplementary Figure S3, Table 2). Members of the overall core were mostly dominant in the legumes.

*Fusarium* sp. 2 was more than double in abundance in legumes compared to in sorghum. The only exceptions between crops were the MOTUs Didymellaceae sp. 2 (*Epicoccum*) and an Unidentified MOTU that were dominant in sorghum despite a few MOTUs, such as Didymellaceae sp. 1 (*Phoma*), also having high abundances in sorghum. Other MOTUs, such as Davidiellaceae sp. (*Cladosporium*) and *Cryptococcus,* oscillated in occurrence and distribution between the crops.Within a crop, MOTU abundances varied between the years, with the majority of them showing a decline across the years.

The PCoA analysis revealed that sorghum was quite distinct from the legumes in both years (Figure 1b). This was evident with the dominance of most core MOTUs in the legume crops (Figure 2). For example, Didymellaceae sp. 1 (*Phoma*), *Fusarium* sp. 2, and an Unidentified MOTU (Ascomycota) were mostly dominant in legumes across the years. Although RA values varied between crops, MOTUs were still consistently present in all of the crops. For instance, Didymellaceae sp. 1 (*Phoma*) were highest in cowpea, with an average abundance across years of 18.4% (compared to sorghum, which had an average abundance of 11.2%), and *Fusarium* sp. 2 had the highest average RA in soybean of 13.9% (while lowest in sorghum, with 4.1%).

Within each crop, Bambara groundnut had 10 core MOTUs present in all substrates between the years, followed by soybean (9 MOTUs), sorghum (9 MOTUs), dry bean (8 MOTUs), and cowpea (7 MOTUs) (Figure 2). The core MOTUs varied in RA between the years, while some were relatively the same. For example, Didymellaceae sp. 1 (*Phoma*) was mostly dominant in relative abundance in year one. Other MOTUs, such as Davidiellaceae sp. (*Cladosporium*) and *Cryptococcus,* were higher in terms of RA in year two. Didymellaceae sp. 2 (*Epicoccum*) and *Fusarium* sp. 2 were relatively the same in relative abundance between the years. For example, Didymellaceae sp. 1 (*Phoma*) overall was eight time more in relative abundance in year one compared to year two.

A number of unique MOTUs per crop were detected (Figure 2; Supplementary Figure S3). Sorghum had unique MOTUs assigned as *Rhizopus* and Pleosporales, with the Pleosporales MOTU also being core in soybean. For Bambara groundnut, the unique cores consisted of *Mucor* and unidentified MOTUs in the Sordariomycetes and Diaporthales with the Sordariomycete MOTU also present in dry bean. In soybean, the unique MOTU *Bipolaris* occurred in low relative abundances.

### 3.4. Core Mycobiomes of Plant Tissues and Soils

Between plant tissue and soil substrates, the distribution of fungal core showed some differences. Davidiellaceae sp. (*Cladosporium*), Didymellaceae sp. 1 (*Phoma*), Didymellaceae sp. 2 (*Epicoccum*), and an Unidentified MOTU (Ascomycota) had higher RA in plant tissues, and in some instances had RA more than double that compared to soils. For example, Didymellaceae sp. 1 (*Phoma*) occasionally had RA 10 times higher in the plant tissues than in the soils. *Fusarium* sp. 1 varied between the plant tissues and soils, albeit the plant tissues possibly showing dominance. This MOTU was assigned in the pipeline as *Nectriaceae* but shown in [37] to represent *Fusarium*. *Cryptococcus* was relatively balanced between the substrates.

**Figure 3:**
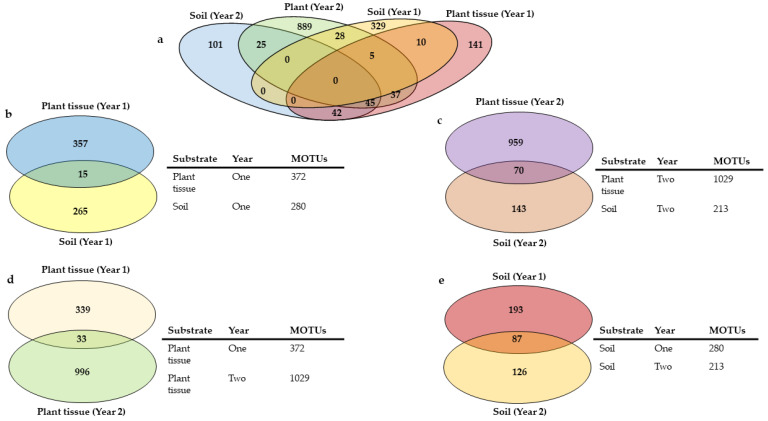
Numbers of unique and shared fungal MOTUs in combined plant tissues and soils over two years overall (**a**) as well as plant tissue and soil of year one (**b**), plant tissue and soil of year two (**c**), plant tissue from year one and two (**d**), and soils from year one and two (**e**).

Plant and soil samples grouped separately in PCoA analyses (Figure 1a), with the soils showing much more variation between the years, while the plants were more stable with overlaps between the years. The plant and soil cores were statistically significant (*p* = 0.001), despite sharing a common core (Table 3; Figure 3a–c). Plants were more diverse than soils in both years, with the diversity of soil more or less the same and in plants increasing by three times in the second year (Figure 3b,c). The overlap between plants and soils increased more than four times in year two. Within substrates, variations were also observed between the years, but more within the plant tissues (Figure 3d) than the soils, which were relatively the same (Figure 3e). The degree of overlap between years for plants and soils was less than the number of unique MOTUs.

Generally, the core MOTUs varied in abundance between the plant tissues and soils, with most decreasing across the years (Table 2). The RA of the Davidiellaceae sp. (*Cladosporium*) mostly decreased across the two years between substrates, except in sorghum where the MOTU was similar in the plant tissues and increased from year one to year two in the soils. Didymellaceae sp. 1 (*Phoma*) showed a similar trend in both plant tissues and soils except in cowpea plant tissues, for which the MOTU increased in year two. Didymellaceae sp. 2 (*Epicoccum*) decreased in RA across years in both plant tissues and soils across years despite the abundances being similar in some of the soils. *Fusarium* sp. 2 increased in abundance in the plant tissues across years, while in soils, it was quite the opposite, with the MOTU decreasing. *Cryptococcus* increased in abundance from year one to year two in both plant tissues and soils.

Plant tissues and soils were observed to have unique MOTUs across the years (Table 3). The unique MOTUs in plant tissues included MOTUs that were not assigned to genus level in the Pleosporales and Sordariomycetes. The soils were observed to include *Bipolaris*, *Mucor*, and unidentified MOTUs in the Dothideomycetes and Hypocreales that were not assigned to a genus. In the plant tissues, Pleosporales were relatively similar across the years, except for sorghum plant tissues. The Sordariomycetes MOTU fluctuated in the plant tissues across years with high abundances in year one for Bambara groundnut and in year two for cowpea and soybean. In some instances, it was similar between the years. With the soils, the RA of *Bipolaris* was relatively similar across the years. The MOTU in the Hypocreales increased in the soils, with a value of more than 15% in year two. *Mucor* increased across years in sorghum and Bambara groundnut while with the other soils, the abundances were relatively similar.

### 3.5. Core Mycobiomes across the Years

Several distinct patterns were observed with statistical significance (*p* = 0.001 ***) between the years with regards to substrates and crops (Table 3; Figure 1). Mycobiomes of plants in year two were more variable, (Figure 1b) while in their soils, year two was more homogenous (Figure 1c). The occurrence of the MOTUs differed, with some MOTUs present in both years while others were unique to a year in a substrate or crop (Table 2 and Table 3, Figure 1 and Figure 3). The overall core mostly decreased in relative abundance between the years in substrates and between crops. For instance, the Didymellaceae sp. (*Phoma* and *Epicoccum*) generally decreased in relative abundances in the substrates between the years. These MOTUs also showed a similar trend between years in crops. Other MOTUs, such as *Cryptococcus,* increased in relative abundance between the years, and a similar trend was observed within the substrates from year one to year two. Over the years, in crops, the MOTU increased, but the greatest increase was in dry bean, which was almost 10 times higher in year two.

Some MOTUs were unique to a substrate between the years (Table 3). The plant tissues were less variable in terms of the occurrence of unique MOTUs in both years compared to soils, which had more unique MOTUs in year two. In year one, the number of unique MOTUs was slightly lower than in year two. In year one, the prominent unique MOTUs included *Geomyces* and *Myrothecium*, while for year two included *Fusarium* sp. 3 (assigned in the pipeline with an old teleomorph name of *Fusarium*, *Gibberella*) and *Mucor*. In the soil, the year-one soils had a greater number of unique MOTUs compared to year two. Those that were prominent in year one included the *Geomyces*, *Fusarium* sp. 4 (assigned in the pipeline with an old teleomorph name of *Fusarium* as *Haematonectria*), and *Mortierella*. Year two included prominent MOTUs, such as *Diaporthe*, *Phomopsis,* and *Fusarium* sp. 1.

## 4. Discussion

In this study, the composition of the core mycobiome within plants and the soil environment in a cereal–legume intercropping system grown over a two-year period was explored. Generally, the core mycobiomes of plant tissues and soils were quite distinct. Robust core communities existed for the crops consisting of sorghum, Bambara groundnut, soybean, cowpea, and dry bean that was ubiquitously present for both year one and two. Most of the components of the core mycobiomes varied in abundance and occurrence between the crops and substrates across the years.

Several patterns in abundances of core fungal MOTUs were observed. Sorghum was quite different in abundances in some of the core MOTUs when compared to legumes. Year-two plants were more variable than those in year one. This could have been due to the residual effect of fungi acquired during the first year upon planting of crops in year two. The soils in year two were more homogeneous in, possibly due to an intercropping effect after two years. Intercropping could have contributed to changes in abundances of MOTUs between the crops, which favours development of different types of roots and distribution in the soil changing the exudation process in the rhizosphere, influencing abundance and interactions between plants and microorganisms [41,42]. Future studies across more years and more locations are essential to investigate these trends.

Within the soil environment, a slight decline in the number of MOTUs was observed from year one to year two, yet communities remained distinct based on the PCoA analysis. The trend we observed with the soils between years one and two contradicts those of previous reports. Other studies usually detected an increase in fungal community diversity in soils with intercropping and increased plant diversity for two years or more, thus improving nutrient availability or facilitating niche partitioning, water uptake, and organic matter decomposition for healthier soils, and stimulation of plant health and productivity [43,44,45,46,47,48]. However, this study did not monitor changes in soil communities from a monoculture to an intercropping plot but monitored changes between two years of intercropping.

The relative abundances of the core mycobiome components varied between the two years, with most of them decreasing across the years. Those which declined included MOTUs assigned to *Cladosporium* (Davidiellaceae) and *Phoma* (Didymellaceae), which were commonly associated with all the substrates in both years. In contrast, MOTUs assigned to *Fusarium*, which increased more prominently in year two, especially in the plant tissue and soils. Their abundance was largely high in all the plant tissue of legumes, but for sorghum, *Fusarium* was more dominant. Mycobiomes which were unique were sporadically detected between the years, crops, and substrates. For example, the assigned MOTU Diaporthales in the cores and other assigned MOTUs in *Bipolaris* and *Curvularia* were only core to soils. The fluctuations between the years may indicate that plants and different substrates play an important role in the proliferation of sorghum-legume associated fungal communities. However, data from more years should be added to determine if these are not only natural fluctuations.

The overall core MOTUs in the cereal–legume intercrop were comprised of well-known plant-associated taxa [49,50]. The overall core MOTUs that were detected for substrate, crop, and years included Davidiellaceae (assigned in the pipeline as *Cladosporium*), Didymellaceae (assigned in the pipeline as *Phoma* and *Epicoccum*), four *Fusarium* MOTUs, and *Cryptococcus*. These taxa have previously been reported to be part of core mycobiomes in other crops, such as rice, sugarcane, wheat, and *A. thaliana* [23,25,26,51]. The presence of these fungal communities as core could suggest their successful adaptation to different environmental and agricultural factors within the phytobiome, as well as adaptation to life on or within plant tissue and soils. Furthermore, their presence and difference in abundance between the crops could have been as a result of plant-specific selective factors which have an effect on community structure [52,53]. Reference [54] also reports that although plants can be of a different species or genotype when grown in the same location, they largely form a common core microbiota because of the same environmental “inoculum”.

*Fusarium*, with older names *Giberella* and *Haematonectria* [55], was a key genus in the plant tissues and soils for each crop in both years, with greater abundance in year-two plant tissues. *Fusarium* increased between the years but was more abundant in legumes compared to sorghum. A possible explanation could be that these *Fusarium* MOTUs successfully established themselves in the first year and carried over to the second year, increasing in abundance, resulting in possible horizontal transmission of spores from the soil into the plants when new plants were established. Species in *Fusarium* are of fundamental importance and are commonly isolated from various substrates which include soil, water, air, and dead plant material. Some are able to colonize plant tissue as endophytes, acting as biocontrol agents against other plant pathogenic fungi or becoming devastating plant pathogens and contaminants in agricultural produce (mycotoxins) [56,57]. Species present in this study could thus have different roles. A recent study comparing soils of seven intercropping setups, which included cucumber (*Cucumis sativus),* alfalfa (*Medicago sativa*), trifolium (*Trifolium repens*), wheat (*Triticum aestivum*), rye (*Secale cereale*), chrysanthemum (*Chrysanthemum coronrium*), rape (*Brassica campestris*), and mustard (*Brassica juncea*), revealed that *Fusarium* was also among the common genera found in the soils [58].

The MOTU in the family Davidiellaceae (assigned by the pipeline as *Cladosporium*) was detected in all the plant tissue and soils for each crop in year one and two, but was mostly prominent in plant tissue, especially in the first year. Species in Davidiellaceae, including those in *Cladosporium*, are widely distributed cosmopolitan fungi, commonly isolated from the soil, plant, food, and organic matter/debris [59]. Some species in *Cladosporium* are known plant pathogens, causing leaf spots and other lesions [60,61,62]. However, some *Cladosporium* species, such as *Cladosporium cladosporioides* and *C. pseudocladosporioides*, have biological control properties that cause them to parasitize other fungi, such as *Puccina horiana* (Henn), the causal agent of chrysanthemum white rust [63]. Some species are commonly known endophytes as well as phylloplane fungi [59]. *Cladosporium* has been found to be core in the outer compartments of the seed of six different rice cultivars [22]. The Davidiellaceae (*Cladosporium*) was found to be one of the key species in the soils of seven intercropping systems, involving one common crop and several different crops per system [58].

The Didymellaceae (assigned in the pipeline as *Phoma* and *Epicoccum*) was also identified in all plant tissues and soils of each crop for both years of the intercropping system, with the family mostly prominent in the plant tissue. *Phoma* and *Epicoccum* species are widely distributed and found in diverse ecological niches, such as plants and soil [64,65]. They are known to be opportunistic fungi but can be pathogenic when they colonize and establish in the right plant host [65,66]. Some species are known pathogens of economically important crops including wheat, sorghum, and soybean [67].

*Cryptococcus* was core in all of the plant tissues and soils and is known to be ubiquitous in nature. Species in *Cryptococcus* have also been reported to be endophytes isolated and detected in the living tissue of plants i.e., leaves, stems, and roots [68,69]. In a previous study, *Cryptococcus* was among the common genera associated with the leaves and stems of vascular plants (*Cassiope tetragona*, *Saxifraga cespitosa*, *Saxifraga oppositifolia*, and *Silene acaulis)* in the high arctic zones of Ny-Ålesund region, Svalbard [69]. Recent studies have reported that *Cryptococcus* is also a dominant fungal genus in soils [70,71]. Some species have positive biological properties reported on plants, such as growth promotion [72]. Other than plants, some *Cryptococcus* species found in the environment are known fungal pathogens in humans, causing opportunistic infections [73].

Other fungal groups that were unique and specific cores to a year or a specific substrate were also observed. This suggests their presence in specific substrates and years could have been a result of opportunistic infections and establishments due to favourable conditions [26,74,75]. These included *Geomyces* and *Myrothecium Paecilomyces*, *Penicillium, Fusicolla, Stachybotrys,* and *Fusarium* sp. 1 and 4, *Fusarium* sp. 1, *Bipolaris, Curvularia*, and *Mortierella*. Furthermore, the plot trial was surrounded by other trials/plots, which included other crops—e.g., maize and sunflowers—which could have contributed to possible opportunistic infections and establishment of some of these fungi in specific niches or years.

Numerous MOTUs that could not be assigned to a family level were detected as core mycobiomes and varied in diversity between the years and niches. These included MOTUs in the Sordariomycetes, Pleosporales and Diaporthales, and Unidentified MOTUs that could not be assigned to orders and families. These sequences were assigned to fungal MOTUs that are not well represented in the UNITE database due to lack of taxonomic classification and rank, and undiscovered taxa [27,76]. Although there are challenges in the resolution of unidentified classifications [77], their presence also indicates that further analysis is needed to characterize their diversity and possible roles down to genus levels. Therefore, the presence of the unassigned MOTUs suggests that further research must be conducted to accurately characterize and determine their role and presence in the core mycobiome. Species in the Pleosporales, Diaporthales, and Sordariomycetes are saprobes, beneficial, and plant pathogen endophytes and epiphytes associated with plants, respectively [78,79].

A major limitation in sorghum production is stalk and root rot, which is mainly associated with a variety of soilborne fungi such as *Fusarium* species, *Macrophomina phaseolina*, *Pythium* species, and *Colletotritichum graminicola* [80,81,82,83]. These genera are also problematic in legume production [84,85]. Infection can cause major yield losses in the field and also reduce grain quality and contamination during storage. MOTUs assigned to *Fusarium* were detected in the plant tissue and soils of the cereal–legume intercrop in high abundances, especially in year-two plant tissue and in some soils in year one. Although it was not established whether the MOTUs assigned to *Fusarium* are those belonging to the pathogenic group causing stalk and root rot, amplicon sequencing did detect an increase in MOTUs associated with the genus. Further research must be conducted to accurately identify MOTUs linked to these known pathogenic groups.

## 5. Conclusions

In this study, it was found that in an intercrop consisting of sorghum and four legumes, a core of dominant MOTUs existed that was shared by both the cereal and the legumes. These core mycobiomes consisted of similar genera as those found for other crops in other parts of the world. Components of the core mycobiome of the plants studied here were also shared by the core mycobiomes of the surrounding bulk soils. Fluctuations of these core communities between crops, plant and soil substrates, and years occurred in the relative abundances of MOTUs and their presences and absences. Agricultural practices, such as intercropping, play a key role in plant health and productivity in agricultural systems [43] and have been linked to the plant microbiome and its functionality in various biological processes and plant interactions [17]. Should these core mycobiomes be used to improve performance of sorghum and legumes grown in an intercropping system as well as to increase resistance to disease and overall soil health, it should be established how the core mycobiome can be influenced and if that is possible. Benefits linked to such studies could help in improving productivity of sorghum and underutilized crops, such as Bambara groundnut and cowpea, which would greatly aid in increasing food security, especially in Africa.

## Figures and Tables

**Table 1:** Sequencing results and molecular operational taxonomic units (MOTUs) of plant tissues and soils for year one and two of a sorghum–legume intercrop system.

	Crop	Year	Number of Reads	Total MOTUs
Plant tissues	Bambara groundnut	One	9470	77
	Dry bean	One	8923	87
	Soybean	One	7720	72
	Cowpea	One	8019	66
	Sorghum	One	2098	60
	Bambara groundnut	Two	357,819	185
	Dry bean	Two	679,123	225
	Soybean	Two	845,135	179
	Cowpea	Two	214,228	212
	Sorghum	Two	165,487	229
Soils	Bambara groundnut	One	77,709	747
	Dry bean	One	66,207	364
	Soybean	One	120,151	277
	Cowpea	One	50,611	791
	Sorghum	One	139,545	277
	Bambara groundnut	Two	77,709	155
	Dry bean	Two	76,769	229
	Soybean	Two	74,069	203
	Cowpea	Two	57,655	148
	Sorghum	Two	75,901	272

**Table 2:** Summary of the relative abundance (%) of core molecular Operational taxonomic units (MOTUs) that were assigned in plant tissue and soil, year one and year two in the sorghum-legume intercrop. The bold numbers illustrate abundance greater than 1%.

			Sorghum		Bambara Groundnut		Cowpea		Dry Bean		Soybean	
Niche	Phylum	Core MOTUs	Year 1	Year 2	Year 1	Year 2	Year 1	Year 2	Year 1	Year 2	Year 1	Year 2
Plant tissues	Ascomycota	Davidiellaceae sp. (*Cladosporium*)	**4.9**	**4.8**	**4.5**	0.1	**3.7**	0.6	**9.5**	**6.2**	**3.0**	**0.7**
		Didymellaceae sp. 1 (*Phoma*)	**29.8**	**8.6**	**31.8**	**12.1**	**24.2**	**31.3**	**28.4**	**14.3**	**37.4**	**3.9**
		Didymellaceae sp. 2 (*Epicoccum*)	**4.8**	**3.8**	**3.3**	**0.3**	**0.8**	**0.1**	**0.8**	**0.1**	**1.7**	**0.2**
		*Fusarium* 2	**5.6**	**7.7**	**2.7**	**11.3**	**2.6**	**3.0**	**5.8**	**33.1**	**7.6**	**15.3**
		Unidentified	**12.9**	**9.0**	**20.9**	**2.4**	**25**	**11.4**	**51.1**	**7.4**	**45.7**	**4.9**
	Basidiomycota	*Cryptococcus*	**3.5**	**4.9**	**1.5**	**6.2**	0.2	**3.7**	0.4	**13.9**	0.2	**2.3**
	Unidentified	Unidentified	**2.5**	**4.0**	1.0	0.6	0.6	**1.7**	**1.6**	**2.0**	0.9	**12.6**
Soil	Ascomycota	Davidiellaceae sp. (*Cladosporium*)	0.3	**3.2**	0.2	0.2	0.1	0.1	**1.0**	0.5	0.3	0.1
		Didymellaceae sp. 1 (*Phoma*)	4.3	**2.4**	**15.6**	**2.3**	**16.7**	**1.6**	**7.6**	**5.4**	3.1	**1.6**
		Didymellaceae sp. 2 (*Epicoccum*)	**0.2**	**0.1**	**0.1**	**0.1**	**0.2**	**0.1**	**0.5**	**0.2**	**0.1**	**0.1**
		*Fusarium* 2	**2.4**	**0.8**	**26.3**	**1.2**	**2.6**	**4.8**	**8.9**	**1.3**	**26.0**	**6.8**
		Unidentified	**1.6**	**0.7**	**3.0**	0.1	3.5	**0.1**	**2.2**	**0.5**	0.8	0.2
	Basidiomycota	*Cryptococcus*	**0.1**	**1.5**	0.3	**7.0**	0.2	**8.5**	1.8	**6.9**	0.1	**2.0**
	Unidentified	Unidentified	**61.2**	**17.3**	**7.3**	**7.9**	**6.7**	**14.5**	**23.9**	**16.7**	**12.3**	**14.5**

Numerical values in bold are relative abundances (RA) greater than 1%. Names are used as signed by the UNITE database.

**Table 3:** Comparison between plant and soil (year 1 and 2) and years according to the Adonis permutation test.

Compared Categories	D.F	Sum of Sqs	R2	*p*-Value
Plant and soil	3	4.626	0.60156	0.001
Years	1	1.6955	0.22048	0.001

## Data Availability

The sequencing data generated in this study are deposited in NCBI with accession numbers.

## Supplementary Materials

The following supporting information can be downloaded at: https://www.mdpi.com/article/10.3390/microorganisms10102079/s1, Figure S1: Trial plan of agricultural plot for year 2. In year 1, the sorghum plots only consisted of cultivar PAN8816; Figure S2: Rarefaction analysis of fungal community richness estimates based on sequences that passed the Phred quality score of 25 for years one and two. For plant tissue, MG23, 24, 25, 26, and 27 are Bambara groundnut, soybean, dry bean, sorghum, and cowpea, respectively, for year 1. CV2 represents sorghum for year two. With soils MG13, 14, 37, 38, and 39 are cowpea, Bambara groundnut, sorghum, soybean, and dry bean, respectively, for year 1; Figure S3: Fungal diversity of the core mycobiome. The bar graphs illustrate the diversity richness in plant tissue and soil across years in, sorghum (a), Bambara groundnut (b), cowpea (c), dry bean (d), and soybean (e).
