# Supplementary material for: Plant and Soil Core Mycobiomes in a Two-Year Sorghum–Legume Intercropping System of Underutilized Crops in South Africa
Source: Microorganisms. 2022 Oct 20;10(10):2079. doi: 10.3390/microorganisms10102079 (PMC9611730; doi:10.3390/microorganisms10102079)
Supplement: Supplementary file 1 [file microorganisms-10-02079-s001.zip › microorganisms-1907098-supplementary.pdf]

Supplementary information

PAN8076W x 2<br>PAN8816 x 2<br>NS5511 x 2<br>**Dry bean x 6**	PAN8076W x 2<br>PAN8816 x 2<br>NS5511 x 2<br>**Cowpea x 6**	PAN8076W x 2<br>PAN8816 x 2<br>NS5511 x 2<br>**Soybean x 6**	PAN8076W x 2<br>PAN8816 x 2<br>NS5511 x 2<br>**Sorghum (8816) x 6**	PAN8076W x 2<br>PAN8816 x 2<br>NS5511 x 2<br>**Fallow x 6**	PAN8076W x 2<br>PAN8816 x 2<br>NS5511 x 2<br>**Bambara groundnut x 6**
NS511 x 2<br>PAN8076W x 2<br>PAN8816 x 2<br>**Fallow x 6**	NS5511 x 2<br>PAN8076W x 2<br>PAN8816 x 2<br>**Sorghum (8816) x 6**	NS5511 x 2<br>PAN8076W x 2<br>PAN8816 x 2<br>**Dry bean x 6**	NS5511 x 2<br>PAN8076W x 2<br>PAN8816 x 2<br>**Bambara groundnut x 6**	NS5511 x 2<br>PAN8076W x 2<br>PAN8816 x 2<br>**Soybean x 6**	NS5511 x 2<br>PAN8076W x 2<br>PAN8816 x 2<br>**Cowpea x 6**
PAN8816 x 2<br>NS5511 x 2<br>PAN8076W x 2<br>**Soybean x 6**	PAN8816 x 2<br>NS5511 x 2<br>PAN8076W x 2<br>**Bambara groundnut x 6**	PAN8816 x 2<br>NS5511 x 2<br>PAN8076W x 2<br>**Fallow x 6**	PAN8816 x 2<br>NS5511 x 2<br>PAN8076W x 2<br>**Dry bean x 6**	PAN8816 x 2<br>NS5511 x 2<br>PAN8076W x 2<br>**Cowpea x 6**	PAN8816 x 2<br>NS5511 x 2<br>PAN8076W x 2<br>**Sorghum (8816) x 6**

Figure S1. Trial plan of agricultural plot for year 2. In year 1, the sorghum plots only consisted of cultivar PAN8816.

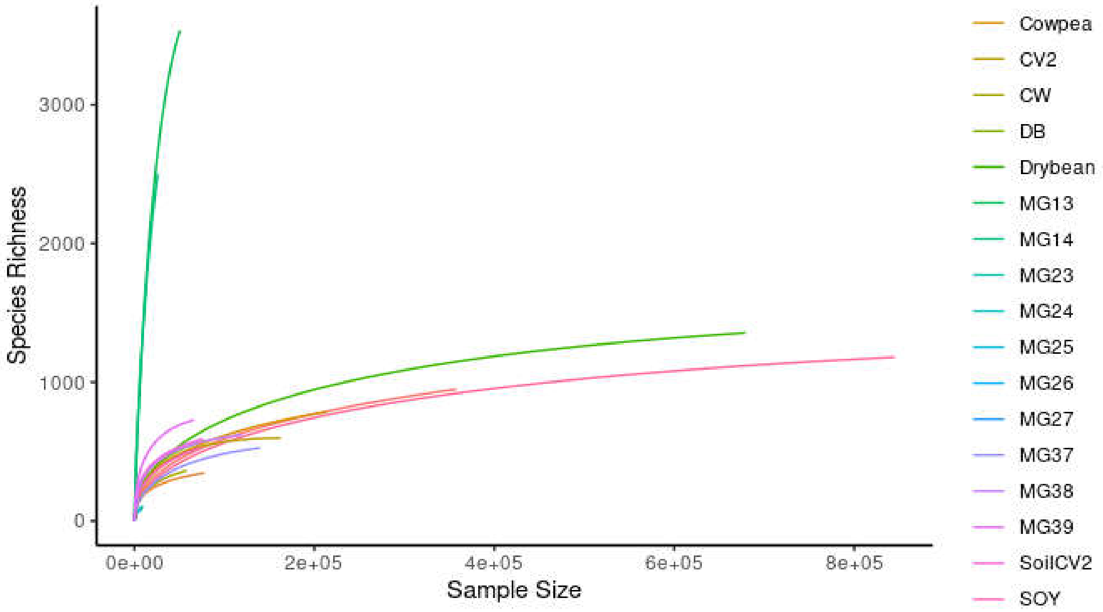

Figure S2. Rarefaction analysis of fungal community richness estimates based on sequences that passed the Phred quality score of 25 for years one and two. For plant tissue, MG23, 24, 25, 26 and 27 are Bambara groundnut, soybean, dry bean, sorghum, and cowpea respectively for year 1. CV2 represents sorghum for year two. With soils MG13, 14, 37, 38 and 39 are cowpea, Bambara groundnut, sorghum, soybean, and dry bean respectively for year 1.

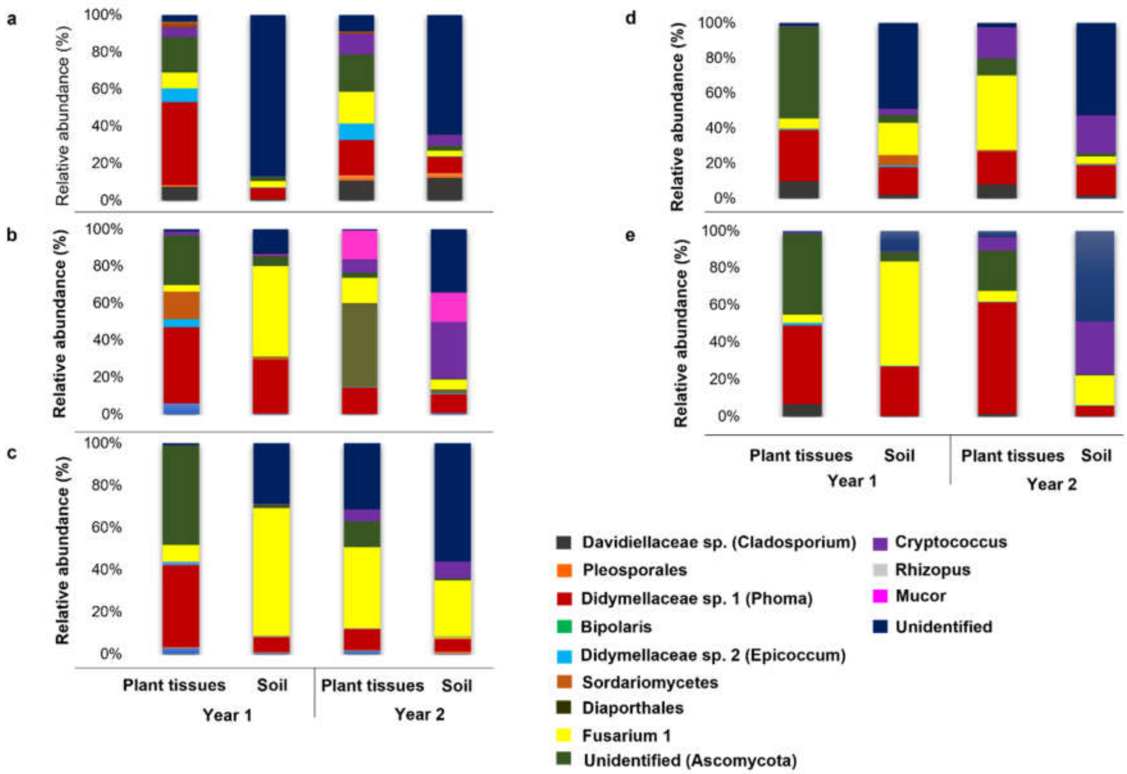

Figure S3. Fungal diversity of the core mycobiome. The bar graphs illustrate the diversity richness in plant tissue and soil across years in, sorghum (**a**), Bambara groundnut (**b**), cowpea (**c**), dry bean (**d**), and soybean (**e**). Raw sequence reads associated with this study generated with Illumina sequencing have been deposited into the Bioprojects depository of Genbank, NCBI. Numbers have not yet been received before the deadline of the special issue. It has been indicated in the text where the project numbers will be inserted.
